# Publisher Correction: LKB1 is the gatekeeper of carotid body chemosensing and the hypoxic ventilatory response

**DOI:** 10.1038/s42003-022-03744-8

**Published:** 2022-07-29

**Authors:** Sandy MacMillan, Andrew P. Holmes, Mark L. Dallas, Amira D. Mahmoud, Michael J. Shipston, Chris Peers, D. Grahame Hardie, Prem Kumar, A. Mark Evans

**Affiliations:** 1 Centre for Discovery Brain Sciences, Hugh Robson Building, University of Edinburgh, Edinburgh, EH8 9XD UK; 2 School of Biomedical Sciences, Institute of Clinical Sciences, College of Medical and Dental Sciences, University of Birmingham, Birmingham, B15 2TT UK; 3 School of Pharmacy, University of Reading, Reading, RG6 6UB UK; 4 Division of Cell Signalling and Immunology, School of Life Sciences, University of Dundee, Dow Street, Dundee, DD1 5EH UK

**Keywords:** Physiology, Neuroscience

Correction to: *Communications Biology* 10.1038/s42003-022-03583-7, published online 29 June 2022.

The original version of this article contained errors in the text.

In the original version of the Article, the third paragraph of the Introduction incorrectly stated “LKB1 is also regulated by direct phosphorylation of eleven of the twelve AMPK-related kinases…”. The is now corrected to, “LKB1 also regulates by direct phosphorylation eleven of the twelve AMPK-related kinases…”

In the Introduction section, page 2 “precipitates” had been changed to “precipitate”, which is now corrected.

In the Results section, page 2, errors were introduced that have now been corrected:

Fig. 1dI, e-g now reads Fig. 1di, e-g

Fig. 1dII, e-g now reads Fig. 1dii, e-g

Fig. 1dIII, e-g now reads Fig. 1diii, e-g

In the Discussion section page 11, “Further support for this proposal is provided by the rank order by the severity” has been corrected to

“Further support for this proposal is provided by the rank order of severity”

In the Discussion section, page 11 a comma has been deleted after “while by contrast,” so that the sentence now correctly reads: “while by contrast the peak of the Sustained Phase of both responses remained unaltered”.

These corrections have been made in the PDF and HTML versions of the Article.

