# Supplementary material for: LKB1 is the gatekeeper of carotid body chemosensing and the hypoxic ventilatory response
Source: Commun Biol. 2022 Jun 29;5:642. doi: 10.1038/s42003-022-03583-7 (PMC9243028; doi:10.1038/s42003-022-03583-7)
Supplement: Supplementary file 3 — Description of Additional Supplementary Files [file 42003_2022_3583_MOESM3_ESM.pdf]

Description of Additional Supplementary Files

File name: Supplementary Movie 1
Description: Hypoxic ventilatory response of control (TH-Cre) mouse.

File name: Supplementary Movie 2
Description: Hypoxic ventilatory response of homozygous Lkb1 knockout mouse.

File name: Supplementary Movie 3
Description: Hypercapnic hypoxic ventilatory response of homozygous Lkb1 knockout mouse.

File name: Supplementary Movie 4
Description: Hypoxic ventilatory response of homozygous AMPKa1+a2 knockout mouse.
