# Supplementary material for: LKB1 is the gatekeeper of carotid body chemosensing and the hypoxic ventilatory response
Source: Commun Biol. 2022 Jun 29;5:642. doi: 10.1038/s42003-022-03583-7 (PMC9243028; doi:10.1038/s42003-022-03583-7)
Supplement: Supplementary file 1 — Peer Review File [file 42003_2022_3583_MOESM1_ESM.pdf]

nature portfolio

Peer Review File

LKB1 is the gatekeeper of carotid body chemo-sensing and the hypoxic ventilatory response

Open Access This file is licensed under a Creative Commons Attribution 4.0 International License, which permits use, sharing, adaptation, distribution and reproduction in any medium or format, as long as you give appropriate credit to the original author(s) and the source, provide a link to the Creative Commons license, and indicate if changes were made. In the cases where the authors are anonymous, such as is the case for the reports of anonymous peer reviewers, author attribution should be to 'Anonymous Referee' followed by a clear attribution to the source work. The images or other third party material in this file are included in the article's Creative Commons license, unless indicated otherwise in a credit line to the material. If material is not included in the article's Creative Commons license and your intended use is not permitted by statutory regulation or exceeds the permitted use, you will need to obtain permission directly from the copyright holder. To view a copy of this license, visit http://creativecommons.org/licenses/by/4.0/.

Reviewers' comments:

Reviewer #1 (Remarks to the Author):

This is an interesting manuscript that reports the findings of an experienced investigative, collaborative team whose area of expertise is hypoxic chemosensitivity, chemo transduction, chemo transmission within cells and neurons in the carotid body and central respiratory network, and pulmonary artery smooth muscle cells. A central theme of much of their work is the role of AMPK key signaling molecule that senses cellular energy and regulates downstream pathways that regulates activity of cells. They have made many novel and substantive discoveries that has advanced our understanding of molecular and cellular mechanisms mediating hypoxic chemosensitivity.

The findings outlined in this current manuscript primarily focuses on the role of the LKB1, signaling molecule which when activated phosphorylates AMPK (and other kinases) then initiates a cascade of phosphorylation of other signaling molecules involved in cellular activation and or adaptation. LKB1 is ubiquitously expressed in essentially all cells and is activated by a variety of stimuli of which AMP+ADP/ATP ratio, a read out for cellular energy, is a major one. AMPK is also ubiquitously expressed in all cells. The authors have performed a series of experiments to differentiate the effect of LKB1 and and AMPK gene deletion on peripheral and central components of the hypoxic ventilatory responses.

The principal conclusion from this current work is that the levels of lkb1 gene expression modifies the hypoxic chemosensitivity of dissociated type 1 cells, chemoafferent responses to hypoxia/hypercapnia, and the hypoxic and hypoxic/hypercapnic ventilatory. They present data from a comprehensive series of experiments interrogating the role of LKB1 and AMPK in the contribution of the carotid body and central (chemoreceptors) on integrated hypoxic ventilatory responses in genetically modified mice. They conclude that LKB1 is functioning primarily at the level of the carotid body and determines a "set-point" about which carotid body afferent discharge is modulated by hypoxia and hypercapnia. However, the gene dose does not appear to be linear and there is divergence from the effect of LKB1 and AMPK gene deletion from the physiologically (cellular and ventilatory) responses to hypoxia. Specifically, it appears that LKB1 site of function is in the carotid body, with gene KO LKB1 of abolishing or markedly attenuating the chemoafferent response to hypoxia and hypercapnia, and attenuating the augmenting phase of hypoxic ventilatory responses.

AMPK is down stream of LKB1, and while deletion of LKB1 in TH expressing cells had significant effect (abolishes) the hypoxic and hypercapnic responses in vitro, AMPK α1+α2 deletion in carotid body type 1 cells increases basal afferent discharge but had no effect on the hypoxic evoked or hypercapnia responses in vitro. LKB1 can phosphorylate other secondary kinases that regulate cellular activity such as CaMkk2, however, CaMkk2 does not appear to affect hypoxic ventilatory responses.

However, in animals with either AMPK or LKB1 gene deletion, breathing is markedly dysregulated. LKB1 gene deletion was associated with, apnea and/or Cheyne-Stokes breathing (CSB) during exposure to severe hypoxia. The frequency CSB was not directly correlated with sighs in animals with deletion of the LKB1 gene deletion. Of importance, CSB could be reversed in these animals by the addition of CO_2 . AMPK KO mice also had prolonged and spontaneous apneas but CSB was not observed (from the best I can tell), and the addition of CO_2 did not reduce the frequency of apnea. These are all novel findings.

The paper has multiple strengths 1) tested a new and novel hypothesis, 2) exploited the power of mouse genetics, 3) used a combination of reduced preparations (cells and tissues), and whole animal physiology to assess the effect the gene deletions. The methods and findings are well described, and while some of the findings are unexpected the experiments appear to be sufficiently done with adequate controls to substantiate and validate the findings.

My most signficant critique is, while the paper is well written, at the beginning of the paper it is difficult to discern what condition is the CONTROL condition; is it the TH-Cre or is it Wild type; and what was the TH-CRE condition. I concluded, only after multiple reads that TH-CRE were animals in whom the a CRE cassette was cloned into the TH promotor and all cells have normal levels of TH expression and LKB1. I assume this is the case, and thus my review is based on this understanding. I think the manuscript would be improved and easier to understand if the figures are arranged such that the TH-CRE responses are compared with the TH- LKB1-homo KO, and wild-type controls; and if the 90% reduction global knock down is of interest, then the comparator

should be to wild type controls.
Comparing the floxed LKB1 where all the cells only express 90% of the gene to the response in the TH/LKB1/KO where 100% of the LKB1 is KO but only in TH catecholamine producing cells, really confuses the picture and distracts from the message; two parameters are changing at the same time: the dose of the gene and the type of cells that are being affected by the deletion. A justification for the design should be provided. While it might be fine to describe what a 90% global reduction in LKB1 gene expression might be doing to the outcomes of interest, the comparator should be the wild-type control where all the cells express 100% of the gene. The authors should also provide evidence that the protein expression for LKB1 is reduced by 90% in the tissue or cells of interest such as carotid body, brainstem regions of interest, or a signaling pathway that is being regulated by LKB1 is reduced by 90%. Perhaps, a more optimal experimental design would have been to create a mouse where 50% reduction in the gene expression. This would allow a better understanding of a "gene dose effect" on the responses described.
Honestly, I am not convinced that the findings in the animals (floxed) with 90% reduction in gene expression really adds a lot to the conclusions of the paper, and I believe that the paper would be much easier to read and follow if the experiments with the "Lkb1-floxed (hypomorphic)" animals were removed because animals with the 90% knockdown and the 100% knockout appear have very similar responses with the exception of the augment calcium transients in the dissociated cells in the figure 1 when there was absence of the hypoxic response in the 100% KO in the catecholaminergic producing cells. And while the authors discuss this unexpected finding, a lot of the discussion is conjecture without supportive experiments. The other difference between the two genetically modified mice is that in the hypomorphic animals, CSB was not observed in response to severe hypoxia while this was the case in the mice with KO of LKB1 in catecholaminergic producing cells. It would be of interest to know the change in the level of protein expression or enzymatic activity of LKB1 in the hypomorphic animals in comparison to the wild-type controls and the KO mice.
Additional data that would enhance the manuscript.
1. Showing how 90% reduction LKB1 gene expression translates to protein expression and or enzymatic activity in the regions of interest, at the very least in the carotid body.
2. If changes in cellular or tissue ATP levels are believed to be the reason for the difference in the in the cellular and physiological responses in the different groups, an assay of cellular respiration or measure of ATP levels would be able to substantiate the proposed mechanisms and should be done.
3. Measurement of metabolic rate during the hypoxic exposure would also be of interest. The drop in ventilation in response to hypoxia in newborn models is associated with a drop in metabolism. Thus the relationship between HVR and metabolic rate would be of considerable interest.
In summary, the hypothesis tested is novel and the experiments are well designed, the data are clearly presented, and the work advances the field of our understanding of key signaling pathways upstream of AMPK that appears to have a profound influence on hypoxic chemosensitivity and ventilatory responses to hypoxia thereby linking metabolism and hypoxic chemosensitivity in the carotid body. Nevertheless, I believe the paper could be enhanced by addressing the items in the critique outlined above. Lastly, the discussion could be reduced by 20%.

Reviewer #2 (Remarks to the Author):

Evans and colleagues studied the contribution of the LKB1 and AMPK activation on the carotid body (CB) chemosensory responses and the hypoxic ventilatory response (HVR) during metabolic stress.

Using one mice model where the gene encoding LKB1 (Stk11) is floxed in a manner that delivers ~90% global hypomorphic LKB1 expression and another model, where LKB1 is deleted from all catecholaminergic cells (tyrosine hydroxylase-Cre), they found that LKB1 determines type I cell responses to hypoxia and the increased CB chemosensory discharge during hypoxia and hypercapnia. Conversely, conditional AMPK deletion in catecholaminergic cells increased basal CB discharges but had no effect on the sensitivity of the CB to hypoxia or hypercapnia. In contrast, the HVR was markedly attenuated by both LKB1 and AMPK deletion. Interestingly, LKB1 knockouts

showed hypoxia evoked hypoventilation and apnoea interspersed by pronounced Cheyne-Stokes-like breathing, while hypoventilation and apnoea, but not Cheyne-Stokes-like breathing, were observed with ~90% LKB1 deficiency and AMPK deletion.

Authors proposed that LKB1 as a novel and essential regulator of O2-sensing and CO2 sensitivity in the CB, and discussed the role played by LKB1 and AMPK in the CB and the HVR. They concluded that LKB1 deficiency and/or energy crises within peripheral and central respiratory networks triggers respiratory distress and Cheyne-Stokes breathing.

This is an interesting and well written Ms., which deal with the role played by LKB1-AMPK in CB chemoreception and in the central respiratory centres. This is an excellent study, performed with appropriate methods, and provide clear data. In summary, it is a good contribution to the field. However, there are some issues that need to be revised to further improve the Ms.

The article was written for specialist. No information about the carotid body and its role in cardiorespiratory regulation including HRV is provide in the Introduction section. Moreover, the appropriate references for the CB are quoted late in the text. For a better understanding of a broad audience, it is necessary to reformulate the introduction. What is the rationale of this study?

It is necessary to clarify the hypothesis and how authors will test it. It is not clearly stated in the Introduction the use of a variety of genetic mouse models, along with both in vitro and in vivo approaches. Authors need to state why they used a mice line (LKb1) that show a 90% reduction of LKB1 expression and a conditional homozygous LBK1 knockout mice in the introduction and not in Results.

Authors concluded that the level of LKB1 expression "determines a set point about which type I cells are activated by hypoxia and hypercapnia, and that membrane depolarisation and exocytotic transmitter release" and "LKB1 determines, independent of AMPK, a set-point about which CB afferent input responses are delivered during hypoxia and provide further strong support for our previous proposal that LKB1-AMPK signalling pathways facilitate the HVR at the brainstem". However, an alternative explication of their results is that genetic ablation of LKB1 may impair oxidative phosphorylation and type I cell function as is shown in several other systems (Jessen et al BBC. 1802, 593-600, 2010; Cheg et al. BBA. 1866, 2020; Germain et al. Human Mol Genetics. 22: 952-962, 2013; He et al. Proceedings of the National Academy of Sciences 114 12542-12547, 2017, etc..).

Why LKB1 ablation reduced both CB responses to hypoxia and hypercapnia? Lahiri and Mulligan showed that oligomyn, a metabolic poison that impair oxidative phosphorylation, reduced -or even blocked- the CB chemosensory response to hypoxia without altering the hypercapnic response, but large doses of oligomycin also block the hypercapnic response. Perhaps, CB stimulation with NaCN in genetic ablated LKB1 mice will help to understand the if LBK1 play a specific role in CB chemoreception, or if its absence produces an unspecific effect on cell metabolism.

The discussion on the role played by the CB and brainstem structures (peripheral and central stimulus interactions) in Cheyne-Stokes-like breathing patterns is too long and speculative. What are the brainstem centres implicated in Cheyne-Stokes breathing? What is the role played by other TH neurons implicated in respiration (i.e., Petrosal neuros, etc,)?

To comply with current standard of transparency, the authors should report exact P values for all main analysis (ANOVA) and specific signs to report the strength of the P values in figures. Data in histograms should show all individual values, and all data should be presented as mean +/- SD instead than mean +/- SEM. Please include the individual data in the graphs to allow to see the samples variability, and instead of SEM use SD in the graphs. Thus, please include the individual data in the graphs to allow to see the samples variability, and instead of SEM use SD.

Please explain why is surprisingly, that "both hypoxia- and potassium-evoked calcium transients were markedly augmented in Lkb1 floxed mice (n = 11; Fig 1Dii and E-G), even though these mice were hypomorphic with ~90% lower expression of LKB1 globally when compared to wild type controls" (lines 132-134).

Reviewer #3 (Remarks to the Author):

The present manuscript tested the role of LKB1 on carotid body chemosensitivity and hypoxic ventilatory response. The authors used LKB1 floxed mice and conditional LKB1 Knockout in the carotid body type 1 cells to evaluate carotid body chemosensitivity and hypoxic ventilatory responses and compare the results with AMPKα1+α2 knockouts and AMPKα1+α2 floxed mice. The authors found that LKB1, but not CaMKK2, is required for hypoxic ventilation response, and reveal that LKB1 expression has a role in establishing carotid body function and chemosensitivity. Moreover, they found divergent role for LKB1 and AMPK between the carotid body and the hypoxic ventilatory response. They also found that LKB1 deficiency but not AMPK in catecholaminergic cells precipitates Cheyne-Stokes-like breathing patterns during hypoxia.
The manuscript provides new information to the field, however sometimes information throughout is difficult to follow. Authors should make an effort to make the information of the manuscript clearer.
I have several comments for the authors:

- Please plot individual data (individual points) in graphs to show data variability or plot mean values with SD.
- The range of age of the animals used in the present project is quite big – 3 to 12 months - and is described that age modify carotid body function and carotid body afferent discharges. Was the age of the animals matched between groups? If not, some differences might be related with age.
- The authors postulate that the level of LKB1 expression determines a set point of carotid body activation regulating ATP levels. A very good way to prove this would be to measure nucleotides levels within the carotid body of LKB1 floxed and KO mice. Also, are the levels of ATP released from the carotid bodies in response to hypoxia in these animals?
- Please show RT-PCR for CBs from LKB1 floxed animals in figure 1. It is important to confirm the low expression of LKB1 in LKB1 floxed animals.
- In figure 2 from what I can understand from the electrophysiological typical recordings, LKB1 deficiency seems to also affect the latency time of the hypoxic response both in LKB1 floxed mice and KO, i.e, the time needed to initiate a response. Could the authors plot these results? This can bring additional information in relation to the LKB1 role in the coupling mechanisms.
- Figure 3, can the authors show the typical recordings in LKB1 floxed animals? Also, in this figure the amplitude of the action potential in LKB1 hom KO is higher, in fact seem to double the amplitude of TH-CRE animals. What would be the meaning of this? Could the action potentials be more synchronous in LKB1 hom KO and each action potential be the sum of 2?
- Figure 4, why did not compare the data in AMPKα1+α2 floxed and KO mice with TH-CRE mice? If authors also include here the data from TH-CRE mice it would be clearer that in fact AMPKα1+α2 hom KO did not show an increase in carotid body afferent discharge, yet AMPKα1+α2 floxed mice show a decrease in carotid body afferent discharge in comparison with TH-CRE. Also, could the authors show RT-PCR to confirm the low expression and deficiency of AMPKα1+α2, respectively in AMPKα1+α2 floxed animals and KO.
- Why in Figure 5 the authors compare LKB1 hom Fx with C57/BI6 animals and not with TH-CRE as in the previous figures.
- In the figure 6 can the authors plot minute ventilation. Can also the figure title reflects the AMPK data

Minor
- Figure 1 E-G, describe the colors of bar graphs in figure legend or place above the graphs.
- Figure 2, I believe that the time scale in A must be wrong. Please verify.
- Figure 7, please include time scale in the recordings
- Did the authors made statistical analysis in supplementary figure 2
- In methods section please describe the duration of hypoxia
- Why authors used hyperoxia (95%+5%CO2) as normoxia in calcium experiments and for afferent discharge.
- Please include in statistical analysis in methods section if mean values are provided with SEM or SD.

Rebuttal to Reviewer Comments

Author responses in bold - note references numbers in rebuttal relate to reference list at the end of the rebuttal and thus differ from numbers quoted in the paper.

Reviewer #1 (Remarks to the Author):

We thank this Reviewer for their kind comments regarding the strength and novelty of this manuscript.

1. *My most signficant critique is, while the paper is well written, at the beginning of the paper it is difficult to discern what condition is the CONTROL condition; is it the TH-Cre or is it Wild type; and what was the TH-CRE condition. I concluded, only after multiple reads that TH-CRE were animals in whom the a CRE cassette was cloned into the TH promotor and all cells have normal levels of TH expression and LKB1. I assume this is the case, and thus my review is based on this understanding.*
On re-reading our manuscript it is evident to us that there was far too limited discussion of our choice of controls and in consistency in the use of wild type and TH-Cre mice as controls in Figure 5 adds to the confusion. The manuscript has been revised significantly to address these concerns.

See Page 7, paragraph 2, lines 139-143:
We next examined the impact of LKB1 deletion on carotid body type I cell function. To this end we employed TH-Cre mice as the control group, because these mice were used to deliver LKB1 deletion in catecholaminergic cells and there was no significant difference between the hypoxic ventilatory response of these mice when compared to the background strain (C57/BL6; **Supplementary Fig 2**).

2. *I think the manuscript would be improved and easier to understand if the figures are arranged such that the TH-CRE responses are compared with the TH- LKB1-homo KO, and wild-type controls; and if the 90% reduction global knock down is of interest, then the comparator should be to wild type controls.*
As there is no significant difference between the HVR of C57/BL6 and TH-Cre we decided to avoid repetition and confusion by highlighting this fact and then proceeding with one control as the comparator, namely TH-Cre.

3. *Comparing the floxed LKB1 where all the cells only express 90% of the gene to the response in the TH/LKB1/KO where 100% of the LKB1 is KO but only in TH catecholamine producing cells, really confuses the picture and distracts from the message; two parameters are changing at the same time: the dose of the gene and the type of cells that are being affected by the deletion. A justification for the design should be provided.*
A justification for the design of the experiment is now provided in the Introduction.
Page 5 last paragraph through page 6 first paragraph, lines 106-114:
We set out to examine the mechanism by which AMPK is regulated in the context of the HVR. To this end we employed a three-point assay to assess the relationship between the level of LKB1 expression, carotid body activation during hypoxia and the HVR, by utilising a mouse line which exhibits ~90% global hypomorphic expression of the gene that encodes LKB1 (*Stk11*, hereafter referred to as *Lkb1*)[1, 2] and a conditional homozygous LKB1 knockout mouse line derived from this in which 100% *Lkb1* deletion is triggered by Cre expression via the tyrosine hydroxylase (TH) promoter, which restricts *Lkb1* deletion to catecholaminergic cells including therein carotid body type I

cells. We compared outcomes to those observed in mice where CaMKK2 had been deleted globally.

The use of the "three-point assay" is very important to this investigation. The action of 100% LKB1 deletion alone could be interpreted as simply having a non-selective action that leads to abolition of both the chemosensory responses to hypoxia and hypercapnia, i.e., cell viability is compromised. The fact that chemosensory responses to hypoxia and hypercapnia are preserved where there is ~90% LKB1 deficiency is therefore critically important, even more so given that the degree of attenuation is greater for the response to hypoxia than for the response to hypercapnia.

4. *The authors should also provide evidence that the protein expression for LKB1 is reduced by 90% in the tissue or cells of interest such as carotid body, brainstem regions of interest, or a signaling pathway that is being regulated by LKB1 is reduced by 90%.*
The carotid body in humans is the size of a grain of rice and is very much smaller in the rat. In the mouse it is smaller still and very poorly defined, where the carotid body is a relatively diffuse array of cells that sit within the connective tissue that lies between the SCG and the carotid bifurcation (See Fig 1). The small quantity of tissue is prohibitive when it comes to Western blot.
We have, however, completed qRT-PCR to demonstrate LKB1 deficiency here, and highlighted the caveat that one cannot assume that this reflects the true level of expression in type I cells. Moreover, we have highlighted the fact that others have shown reductions in mRNA and protein in a variety of tissues including the brain.
See page 7 paragraph 1:
Then, the absence of LKB1 expression was confirmed in acutely isolated carotid body type I cells by single cell end point RT-PCR (**Fig 1B-C and Supplementary Fig 1;** consistent with outcomes for other organs including the brain[1, 2], LKB1 expression from homozygous *Lkb1* floxed mice (Ct = 31.147 ± 0.098, n = 3) was lower than for TH-Cre mice (Ct = 25.139 ± 0.006, n = 3) in whole carotid bodies, although it should be noted that this multi-cellular organ is not representative of pure type I cells.

Removing the connective tissue within which the carotid body is located without dragging catecholaminergic cells from the SCG with the it is not a trivial task. Type I cell isolation can be reliably achieved and cross-checked but the carotid body itself cannot be effectively isolated form the connective tissue that surrounds it, or from a variety of other cell types incorporated (e.g, smooth muscle cells and endothelia of blood vessels, neurons, type II cells etc).

5. *Perhaps, a more optimal experimental design would have been to create a mouse where 50% reduction in the gene expression. This would allow a better understanding of a "gene dose effect" on the responses described.*
This would be very useful but is not presently feasible. The LKB1 deficiency is the floxed mice was an unexpected outcome of the gene modification strategy employed. We have taken advantage of this in the present study.

6. *The other difference between the two genetically modified mice is that in the hypomorphic animals, CSB was not observed in response to severe hypoxia while this was the case in the mice with KO of LKB1 in catecholaminergic producing cells.*
This highlights the importance of the "three-point assay" is very important to this investigation. The fact that is not observed where there is ~90% LKB1 deficiency significant.

7. *It would be ofinterest to know the change in the level of protein expression or enzymatic activity of LKB1 in the hypomorphic animals in comparison to the wild-type controls and the KO mice.*
Additional data that would enhance the manuscript.
A. Showing how 90% reduction LKB1 gene expression translates to protein expression and or enzymatic activity in the regions of interest, at the very least in the carotid body.
The amount of tissue and number of animals that would be required to achieve this is prohibitive. See response to point 4.

B. If changes in cellular or tissue ATP levels are believed to be the reason for the difference in the in the cellular and physiological responses in the different groups, an assay of cellular respiration or measure of ATP levels would be able to substantiate the proposed mechanisms and should be done.
The amount of tissue and number of animals that would be required to achieve this is prohibitive. Moreover. To our knowledge, no other laboratory has achieved this in a reliable and accurate manner for mouse carotid bodies.
See response to point 4.
It should be noted, however, that afferent fibre discharge from the carotid body *is the most sensitive assay for exocytotic release of ATP*, which drives afferent fibre discharge through the carotid sinus nerve.

C. Measurement of metabolic rate during the hypoxic exposure would also be of interest.
The metabolic status of AMPK knockout mice has been assessed by AME during normoxia and hypoxia and is normal (unpublished obersvation). However, this detailed study is the subject of a separate manuscript. Others have assessed the metabolic status of the LKB1 floxed mice and reported this to be normal. In addition, we have assessed and now include measures of core body temperature, blood gases and pH in all animal models and now include these data.
See page 10 last paragraph through page 11 first paragraph, lines 222-229 1:
Under normoxia there was no difference between controls and either *Lkb1*, *Camkk2* or, as previously shown[3], dual *AMPKα1+α2* knockouts with respect to weight versus age, breathing frequency, tidal volume, minute ventilation, blood gases, blood pH or core body temperature (**Supplementary Fig 7-8 and Table 1**). Moreover, the metabolic status of *Lkb1* floxed mice[1] and *AMPKα1+α2* knockouts (Evans, unpublished) is normal. Nevertheless, profound genotype-specific differences were observed with respect to the ventilatory responses during hypoxia, and to a lesser extent during hypercapnia.

Reviewer #2 (Remarks to the Author):
We thank Reviewer 2 for their complimentary remarks on our manuscript.

1. *The article was written for specialist. No information about the carotid body and its role in cardiorespiratory regulation including HRV is provide in the Introduction section. Moreover, the appropriate references for the CB are quoted late in the text. For a better understanding of a broad audience, it is necessary to reformulate the introduction.*

We have now reformulated the introduction, which begins with a description of the role of the carotid body in the HVR.
See page 4, paragraph 1 and 2, lines 67-78:

The hypoxic ventilatory response (HVR) delivers compensatory increases in ventilatory drive where there are deficiencies in oxygen availability. The HVR is initiated by increases in afferent fibre discharge from the carotid bodies, the primary peripheral arterial chemoreceptors of mammals that are located within the bifurcations of the carotid artery, ideally situated to monitor blood flow to the brain. Within the carotid body falls in arterial PO_2 (and increases in arterial PCO_2) are sensed directly by carotid body type I cells, where consequent depolarisation elicits exocytotic release of ATP that mediates increases in chemoafferent discharge to the respiratory central pattern generators in the brainstem[4-6].

We recently showed that in addition to regulating metabolic homeostasis in a cell autonomous manner[7] the AMP-activated protein kinase (AMPK) facilitates the HVR and thus oxygen and energy (ATP) supply to the whole body[3]. In doing so, we demonstrated that AMPK acts not at the level of the carotid bodies as one would predict but downstream at the brainstem. Briefly, conditional deletion of AMPK in catecholaminergic neurons of mice precipitates hypoventilation and apnoea during poikilocapnic hypoxia[3], that resembles central apnoea of prematurity[8, 9] and central sleep apnoea[10] in neonate and adult humans, respectively. That said, the HVR of these mice is most reminiscent of the HVR observed in premature infants where the hypercapnic hypoxic ventilatory response is similarly conserved[11]. Given such face validity that resembles symptoms in patients, it is important that we identify the mechanism(s) by which AMPK is regulated in the context of the HVR.

2. *What is the rationale of this study?*

It is necessary to clarify the hypothesis and how authors will test it. It is not clearly stated in the Introduction the use of a variety of genetic mouse models, along with both in vitro and in vivo approaches. Authors need to state why they used a mice line (LKb1) that show a 90% reduction of LKB1 expression and a conditional homozygous LBK1 knockout mice in the introduction and not in Results.

We have revised the Introduction to make this clear.

See page 5, last paragraph through page 6 first paragraph, lines 106-114:

We set out to examine the mechanism by which AMPK is regulated in the context of the HVR. To this end we employed a three-point assay to assess the relationship between the level of LKB1 expression, carotid body activation during hypoxia and the HVR, by utilising a mouse line which exhibits ~90% global hypomorphic expression of the gene that encodes LKB1 (*Stk11*, hereafter referred to as *Lkb1*)[1, 2] and a conditional homozygous LKB1 knockout mouse line derived from this in which 100% *Lkb1* deletion is triggered by Cre expression via the tyrosine hydroxylase (TH) promoter, which restricts *Lkb1* deletion to catecholaminergic cells including therein carotid body type I cells. We compared outcomes to those observed in mice where CaMKK2 had been deleted globally.

The present investigation not only demonstrates that LKB1, but not CaMKK2, is required for the HVR, but also reveals for the first time that the level of LKB1 expression serves a novel and essential role in establishing carotid body function and chemosensitivity. In short, we uncover a divergence in dependency on LKB1 and AMPK between the carotid body on the one hand and the HVR on the other. Adding to this we show that LKB1, but not AMPK, deficiency within catecholaminergic cells precipitates Cheyne-Stokes-like breathing patterns during hypoxia, which are associated with heart failure but of unknown aetiology[12].

3. *An alternative explication of their results is that genetic ablation of LKB1 may impair oxidative phosphorylation and type I cell function as is shown in several other systems (Jessen et al BBC. 1802, 593-600, 2010; Cheg et al. BBA. 1866, 2020; Germain et al. Human Mol Genetics. 22: 952-962, 2013; He et al. Proceedings of the National Academy of Sciences 114 12542-12547, 2017, etc..).*

We thank this Reviewer for highlighting these studies and raising the matter of mitochondrial dysfunction consequent to LKB1 deletion. We had implied that this was the cause of ATP deficiencies in LKB1 knockouts but had not explicitly stated it. Three of these papers are now cited and we state that LKB1 causes mitochondrial dysfunction. We could not find the fourth citation through Pubmed (Cheg BBA 2020).

See page 17, paragraph 2, lines 378-383:

It is intriguing to note, therefore, that previous studies on the effects of *Lkb1* deletion have identified dysfunctional mitochondria and consequent reductions in ATP levels and increases in AMP:ATP and ADP:ATP ratios in a variety of cell types, including skeletal muscle [1], cardiac muscle [2, 13], pancreatic beta cells [14 15], regulatory T Cells[16] and MIN6 cells [14].

4. *Why LKB1 ablation reduced both CB responses to hypoxia and hypercapnia? Lahiri and Mulligan showed that oligomyn, a metabolic poison that impair oxidative phosphorylation, reduced -or even blocked- the CB chemosensory response to hypoxia without altering the hypercapnic response, but large doses of oligomycin also block the hypercapnic response.*

We thank this Reviewer for highlighting this study by Lahiri and Mulligan, which is now discussed. This is a very significant paper that reports on matters that are in accordance with our findings and one of our conclusions drawn, that the carotid body responses to hypoxia and hypercapnia are only partially separate with the response to hypercapnia being partially dependent on mitochondrial function.

See page 9, paragraph 2, lines 192 to 200:

It was a surprise to find, therefore, that *Lkb1* deletion also attenuated carotid body activation during hypercapnia (**Fig. 3A**), given that carotid body responses to hypercapnia are generally presumed to be triggered by ion channel mechanisms independent of mitochondrial metabolism[17-20], although earlier studies had provided pharmacological evidence that pointed to a partial dependence on mitochondria (see Discussion for further details)[21]. CO_2-sensitivity (which is linear between 40 and 80 mmHg) was largely preserved in the *Lkb1* hom flx group (n=6) but was virtually abolished in carotid bodies from homozygous *Lkb1* knockouts (n=4) when compared to TH-Cre (p <0.001, n = 7; **Fig 3B and C, Supplementary Fig 5**).

See page 17, last paragraph through 1st paragraph page 18, lines 394-411:

Although counter-intuitive, a lowering of ATP levels could also explain why hypoxia-evoked calcium transients were augmented in carotid body type I cells with hypomorphic expression of LKB1 yet blocked by homozygous *Lkb1* deletion, if mid-range reductions in basal ATP levels confer increased sensitivity of TASK1/3 channels to inhibition by hypoxia as previously proposed by others[22], without greatly

compromising the capacity for exocytotic ATP release. Further reductions in ATP availability upon homozygous *Lkb1* deletion could then ultimately render these ATP-sensitive TASK1/3 channels inactive, abolish hypoxia-response coupling in type I cells [22-24] and greatly reduce vesicular uptake and exocytotic ATP release[25, 26]. This would also impact on type I cell activation by hypercapnia, due to the fact that this too is in great part mediated by inhibition of TASK1/3 channels through acidosis and consequent induction of exocytotic release of ATP, albeit in a manner independent of mitochondrial oxidative phosphorylation [21]. Indirect support for this proposal may be taken from the previous finding of others that inhibition of mitochondrial oxidative phosphorylation (oligomycin and antimycin A) in cat carotid bodies in-situ blocked responses to hypoxia and enhanced response to hypercapnia *in-vivo*[21]. If LKB1 deficiency did not reduce ATP availability, then one would expect augmented afferent discharge in response to hypercapnia rather than the decreases reported here.

5. *To comply with current standard of transparency, the authors should report exact P values for all main analysis (ANOVA) and specific signs to report the strength of the P values in figures. Data in histograms should show all individual values, and all data should be presented as mean +/- SD instead than mean +/- SEM. Please include the individual data in the graphs to allow to see the samples variability, and instead of SEM use SD in the graphs. Thus, please include the individual data in the graphs to allow to see the samples variability, and instead of SEM use SD.*

We now provide Supplementary Figures that show dot plots of all data points for all experiments reported in our manuscript. We considered using these as the main figures. However, the bar charts originally shown are easier to follow. Therefore, bar charts were retained in the main manuscript.
The use of SEM is retained and sufficient.

Note: **For extracellular recordings of afferent nerve activity all single point experimental data is now shown in Supplemental Figs 4, 5 and 6. A key point to make is that whilst doing this we did spot that whilst the basal discharge in the Lkb1 hom KO group is depleted by approximately 70%, this is not actually statistically significant. We do apologise for this initial oversight and have now made this amendment. The data have not changed and are still highly indicative of a physiologically relevant reduction but we have slightly toned down sections of the results and discussion.**
In the results section it now reads, page 8, paragraph 2, lines 165-171:
By contrast, mean basal afferent discharge from carotid bodies of homozygous *Lkb1* knockouts (n=7) was reduced by approximately 70% (**Fig 2D, Supplementary Fig 4)** although this was not statistically significant (p=0.09 versus TH-Cre and p=0.06 vs *Lkb1* floxed).

6. *Please explain why is surprisingly, that "both hypoxia- and potassium-evoked calcium transients were markedly augmented in Lkb1 floxed mice (n = 11; Fig 1Dii and E-G), even though these mice were hypomorphic with ~90% lower expression of LKB1 globally when compared to wild type controls" (lines 132-134).*

We thank this Reviewer for highlighting this matter, which was not presented well within the text of the results section. This section of the results has now been revised to provide greater clarity.

See page 7, last paragraph through first paragraph page 8, lines 150-157:
Surprisingly, outcomes for type I cells from LKB1 floxed mice and LKB1 knockouts not only different but opposite. Potassium-evoked calcium transients were markedly augmented in *Lkb1* floxed mice (n = 11; **Fig 1Dii and E-G, Supplementary Fig 3**), which are hypomorphic with ~90% lower expression of LKB1 globally when compared to wild type controls[1]. In marked contrast, hypoxia failed to increase intracellular calcium in type I cells from homozygous *Lkb1* knockouts (n = 8), where calcium transients evoked by 50mM potassium chloride were equivalent to controls (**Fig 1Diii and E-G, Supplementary Fig 3**).

Reviewer #3 (Remarks to the Author):

1. *Sometimes information throughout is difficult to follow. Authors should make an effort to make the information of the manuscript clearer.*
With the help of comments from Reviewers 1 and 2 we have completed extensive revision of the manuscript to provide greater clarity of communication (see above). We trust the manuscript is now easier to follow.

2. *Please plot individual data (individual points) in graphs to show data variability or plot mean values with SD.*
We now provide Supplementary Figures that show dot plots of all data points for all experiments reported in our manuscript. We considered using these as the main figures. However, the bar charts originally shown are easier to follow. Therefore, bar charts were retained in the main manuscript.
SEM is retained as this is sufficient.

3. *The range of age of the animals used in the present project is quite big – 3 to 12 months - and is described that age modify carotid body function and carotid body afferent discharges. Was the age of the animals matched between groups? If not, some differences might be related with age.*
We showed data on weight versus age from 2-12 months. Mice were weaned at 2 months. We did now complete plethysmography at this age. Plethysmography was completed between 6 and 12 months, where mice are adults. This is now stated. Adding to this, ventilatory responses were normalised by mouse weight and provided as percentage change.

4. *The authors postulate that the level of LKB1 expression determines a set point of carotid body activation regulating ATP levels. A very good way to prove this would be to measure nucleotides levels within the carotid body of LKB1 floxed and KO mice. Also, are the levels of ATP released from the carotid bodies in response to hypoxia in these animals?*
Afferent fibre discharge from the carotid body *<u>is the most sensitive assay for exocytotic release of ATP</u>*, which drives afferent fibre discharge through the carotid sinus nerve. LKB1 deletion virtually abolished this during normoxia, hypoxia and hypercapnia, while LKB1 deficiency in floxed mice mildly attenuates during hypoxia and hypercapnia but not during normoxia.

The amount of tissue and number of animals that would be required to measure nucleotide levels in mouse carotid bodies from our animal models is prohibitive. Moreover, to our knowledge, no other laboratory has achieved this in a reliable and accurate manner for mouse carotid bodies.

I am sure this Reviewer is aware of the degree of difficulty. The carotid body in humans is the size of a grain of rice and is very much smaller in the rat. In the mouse it is smaller still and very poorly defined, where the carotid body is a relatively diffuse array of cells that sit within the connective tissue that lies between the SCG and the carotid bifurcation (See Fig 1). The small quantity of tissue is prohibitive when it comes to measuring ATP levels in a manner that would identify differences between genotypes. Particularly so given that the carotid body incorporates a variety of other cell types (e.g., smooth muscle cells and endothelia of blood vessels, neurons, type II cells etc). In short, this is not a trivial experiment and has yet to be reliably achieved with reliability for mouse carotid body by us or any other laboratory in the field.

5. *Please show RT-PCR for CBs from LKB1 floxed animals in figure 1. It is important to confirm the low expression of LKB1 in LKB1 floxed animals.*
See page 6 last line through page 7 paragraph 1, lines 132-138:
Then, the absence of LKB1 expression was confirmed in acutely isolated carotid body type I cells by single cell end point RT-PCR (**Fig 1B-C and Supplementary Fig 1;** consistent with outcomes for other organs including the brain[1, 2], LKB1 expression from homozygous *Lkb1* floxed mice (Ct = 31.147 ± 0.098, n = 3) was lower than for TH-Cre mice (Ct = 25.139 ± 0.006, n = 3) in whole carotid bodies, although it should be noted that this multi-cellular organ is not representative of pure type I cells).

Removing the connective tissue within which the carotid body is located without dragging catecholaminergic cells from the SCG with the it is not a trivial task. Type I cell isolation can be reliably achieved and cross-checked but the carotid body itself cannot be effectively isolated form the connective tissue that surrounds it, or from a variety of other cell types incorporated (e.g, smooth muscle cells and endothelia of blood vessels, neurons, type II cells etc).

6. *In figure 2 from what I can understand from the electrophysiological typical recordings, LKB1 deficiency seems to also affect the latency time of the hypoxic response both in LKB1 floxed mice and KO, i.e, the time needed to initiate a response. Could the authors plot these results? This can bring additional information in relation to the LKB1 role in the coupling mechanisms.*
We thank the reviewer for raising this important point and agree that the timings, thresholds, and rates of increase in chemoafferent discharge in response to hypoxia add important information. Responses to hypoxia were measured as the PO_2 was slowly ramped down over an approximate range of 200 to 40 mmHg. To determine a potential left shift/delayed onset we have been able to plot the PO_2 required to achieve a discharge frequency of 5 Hz. 5 Hz is chosen as it lies on the exponential region of the hypoxic response curve but is not of a magnitude at which the discharge is likely to have begun to diminish. These new data have been added for TH-Cre and *Lkb1* hom Flx (Supplementary Fig 4). Indeed, there was a significant left shift/delayed onset in the response curve in the *Lkb1* hom Flx group.

This has been reported in the main results text as follows, page 8 last paragraph thorough to page 9 paragraph 1, lines 177-181:
Furthermore, the PO_2 required to reach a frequency of 5 Hz was lower in the *Lkb1* hom Flx (70±7 mmHg) group compared to TH-Cre controls (96±4 mmHg, p<0.01) (Supplementary Fig 4), indicative of a significant delay/ lower PO_2 threshold for response initiation.

Responses to *Lkb1* hom KO were diminished and did not achieve 5 Hz in 6/7 cases, (see individual data in Supplementary Fig 4B). Although the PO_2 at 5 Hz could be theoretically calculated we did not think it was appropriate to include it for this group.

We have also added in new data for the exponential rate constants (Supplementary Fig 4), which gives information about the rate of chemoafferent frequency increase upon response initiation.

The following text has been added to the results, page 8, paragraph 3, lines 180-181:
Exponential rate constants were consistent between TH-Cre and *Lkb1* hom Flx groups (Supplementary Fig 4)."

Again, as responses were so diminished in Lkb hom KO groups we did not think it was appropriate to include rate constant data for this group.

Similar new data for PO_2 at 5 Hz and exponential rate constants has been added for AMPKα1+α2 hom Flx and hom KO groups and now compared against TH-Cre as requested (Supplementary Fig 6).
Furthermore, we have expanded text in this section to read as follows page 9 last. through page 10 first paragraph, lines 201-214:
In stark contrast, and consistent with our preliminary findings[3], extracellular recordings of single unit activity from the carotid sinus nerve, showed that increases carotid body afferent responses to hypoxia in the *AMPKα1+α2* hom KO group (n=8) remained comparable to TH-Cre (n=8) and *AMPKα1+α2* hom Fx (n = 10) controls; **Fig 4A-C, Supplementary Fig 6;** for single cell PCR see[3]). Basal discharge and peak responses to hypoxia were similar between the 3 groups (**Fig 4A-C, Supplementary Fig 6**). The PO_2 required to reach a frequency of 5 Hz was reduced in the *AMPKα1+α2* hom Fx (73±5 mmHg, $p<0.01$) and showed a similar trend in the *AMPKα1+α2* hom KO group (80±5 mmHg, $p=0.08$) compared to TH-Cre controls (96±4 mmHg) (**Supplementary Fig 6**). There was however, no difference between *AMPKα1+α2* hom Fx and *AMPKα1+α2* hom KO groups. Exponential rate constants were consistent between the 3 groups (**Supplementary Fig 6**). Moreover, carotid body responses to hypercapnia and CO_2-sensitivity remained unaltered in *AMPKα1+α2* hom KO (n=9) compared to TH-Cre (n=7) and *AMPKα1+α2* hom flx (n=9; **Fig 4D-F, Supplementary Fig 6**).

We have also added the following text to the methods. Page 30 last two paragraphs, lines 689-701:
Comparison of the exponential rate constants allowed for determination of any alteration in the rate of increase in chemoafferent frequency per mmHg reduction in the superfusate PO_2, upon hypoxic response initiation. Furthermore, for any given discharge frequency, the corresponding PO_2 could be calculated using the inverse function of the exponential decay curve, as shown below:
$y = (\ln((x - a)/b))/-c$

where y is the *P*O_2 in mmHg, x is the single fibre discharge frequency in Hz and a,b and c are constants as above. Specifically, superfusate *P*O_2 levels were compared when the single fibre chemoafferent discharge frequency was at 5 Hz. This was chosen as it lies on the exponential region of the hypoxic response curve, but is not of a magnitude at which the discharge is likely to have begun to diminish. This method was used to define any *P*O_2 shift in the hypoxic response curve thereby providing information of a potential change in the *P*O_2 threshold required for hypoxic response initiation.

7. *Figure 3, can the authors show the typical recordings in LKB1 floxed animals?*
We thank this Reviewer for pointing this out. An example chemoafferent response to hypercapnia for a *Lkb1* hom Fx carotid body has been added into Figure 3. We agree that this example is important to include and helps to make the figure more complete. We must clarify here that CO_2 responses are only significantly depleted in *Lkb1* hom KO carotid bodies and not *Lkb1* hom Flx. This has been corrected in the text and the raw examples also reflect this.

8. *Also, in this figure the amplitude of the action potential in LKB1 hom KO is higher, in fact seem to double the amplitude of TH-CRE animals. What would be the meaning of this? Could the action potentials be more synchronous in LKB1 hom KO and each action potential be the sum of 2?*
This is an interesting point and we agree that changes in action potential morphology could have important implications in terms of transmitter release in the brainstem and refractoriness. However, we must point out that the electrophysiological recordings in this article are extracellular and therefore cannot be used to make reliable comparisons in AP amplitude. For this we would need to make intracellular recordings from cell bodies in the petrosal ganglion which were not part of this ex vivo preparation. Our preparation is a reliable method to evaluate differences in AP frequency, which is what we have done here. Any differences in amplitude that we do see are more likely due to the position of the extracellular recording electrode in relation to the fiber that is being recorded from. Evaluating the impact of Lkb1 deficiency on AP morphology is however definitely something that we would like to look at in the future. We have now emphasised that these are extracellular recordings by adding the following twice in the results section "Extracellular recordings of single unit activity from the carotid sinus nerve…" We have also changed the sub-title in the methods section to read "Extracellular recordings of carotid sinus nerve activity".

9. *Figure 4, why did not compare the data in AMPKα1+α2 floxed and KO mice with TH-CRE mice? If authors also include here the data from TH-CRE mice it would be clearer that in fact AMPKα1+α2 hom KO did not show an increase in carotid body afferent discharge, yet AMPKα1+α2 floxed mice show a decrease in carotid body afferent discharge in comparison with TH-CRE.*
We thank the Reviewer for raising this point. As requested, *AMPKα1+α2* hom flx and *AMPKα1+α2* hom KO are now compared against TH-Cre in Fig 4. We have redone all the stats as well to take this into account. A key point to note here is that, statistically, the basal activity is not different between the 3 groups. This has been amended in Fig 4, legend title, results text and the discussion. Furthermore, both *AMPKα1+α2* hom fx and *AMPKα1+α2* hom KO groups are left shifted compared to TH-Cre, which is interesting, although they are not different

to each other. As AMPK expression is preserved in the flox group and absent from the hom KO group, the left shift must be independent of AMPK. We are not in a position confirm the reason for the left shift in these circumstances. We maintain that the main emphasis is that any change in ventilation seen in AMPK KO's must be a consequence of actions in the brainstem rather than through substantial and physiologically relevant changes in CB function.
This has been clarified in the discussion. Page 19, paragraph 3, lines 427-433:
We previously reported that dual *AMPKα1+α2* deletion in catecholaminergic cells blocked the HVR, with no discernable effect on carotid body afferent input responses to hypoxia[3]. The data presented here confirm this finding and show that carotid body CO_2 sensitivity remained unaltered following *AMPKα1+α2* deletion. Our present findings do support the idea that severe hypoventilation and apnoea observed during hypoxia in *AMPKα1+α2* mice is due to dysfunction of central respiratory networks rather than any depletion of carotid body activity.

10. *Also, could the authors show RT-PCR to confirm the low expression and deficiency of AMPKα1+α2, respectively in AMPKα1+α2 floxed animals and KO.*
These data are already published. This is now highlighted by citation.

See page 9 paragraph 3:
In stark contrast, and consistent with our preliminary findings[3], carotid body afferent responses to hypoxia in *AMPKα1+α2* knockouts remained comparable to controls (*AMPKα1+α2* floxed mice (n = 5); **Fig 4A-C, Supplementary Fig 6;** for single cell PCR see[3]).

11. *Why in Figure 5 the authors compare LKB1 hom Fx with C57/Bl6 animals and not with TH-CRE as in the previous figures.*
We now compare TH-Cre with LKB1 floxed and LKB1 knockout in Figure 5.

12. *In the figure 6 can the authors plot minute ventilation. Can also the figure title reflects the AMPK data*
Minute ventilaation is whon in Figure 5 for TH-Cre, LKB1 floxed and LKB1 KO.
The title of Figure 6 now also reflects analysis of data for AMPK knockouts. Supplementary Figure 11 shows data for AMPK, which has been published previously in[3].

13. *Figure 1 E-G, describe the colors of bar graphs in figure legend or place above the graphs.*
The colours are explained in Figure 1 by the labels: TH-*Cre*, *Lkb1* Flx and *Lkb1* hom KO.

14. *Figure 2, I believe that the time scale in A must be wrong. Please verify.*
We thank the reviewer for spotting this. The time scale has now been corrected to 100 s in Fig 2A.

15. *Figure 7, please include time scale in the recordings.*
The time scale is now included.

16. *Did the authors made statistical analysis in supplementary figure 2.*
Yes, no significant difference was found. Therefore, none is indicated. This is stated in the results section.
Page 10 last paragraph through page 11 first paragraph, lines 222-229:
Under normoxia there was no difference between controls and either *Lkb1*, *Camkk2* or, as previously shown[3], dual *AMPKα1+α2* knockouts with respect to weight versus

age, breathing frequency, tidal volume, minute ventilation, blood gases, blood pH or core body temperature (**Supplementary Fig 7-8 and Table 1**).

17. *In methods section please describe the duration of hypoxia.*
This is now described in the methods in addition to the results and supplementary data (5min or 10 min).

18. Why authors used hyperoxia (95%+5%CO2) as normoxia in calcium experiments and for afferent discharge.
In isolated type I cell experiments, we used bicarbonate buffered saline, i.e., 5%CO_2 was used to adjust the pH of the solution to 7.2.
We equilibrated the reservoirs of PSS with 95% air to achieve physiological levels of PO_2 in the superfusate. PO_2 levels in our 'normoxic' solution was circa 150mmHg and this was reduced to circa 20mmHg (see attached) following exposure the hypoxia solutions as monitored using carbon fibre electrodes.
Results, Page 7, paragraph 2, lines 145-147:
Hypoxia (mean±SEM *PO_2* = 20.19±1.73mmHg, ~2% O_2 (n=10); from normoxia ~150mmHg, ~21% O_2)

Methods, page 28, paragraph 2, lines 647-651:
Cells were perfused with a solution consisting of (mM): NaCl (117), KCl (4.5), $MgCl_2$ (1), $CaCl_2$ (2.5), $NaHCO_3$ (23), Glucose (10), pH adjusted to 7.2 using 5% CO_2. For *normoxia* perfusate was bubbled with 5% CO_2 95% air (~150mmHg). For *hypoxia* perfusate was bubbled with 5% CO_2, 95 % nitrogen (~20mmHg).

For the isolated carotid body. See Methods, page 30, last apragraph, lines 693-696:
Chemoafferent responses to hypercapnia were induced by raising the superfusate PCO_2 from approximately 40 mmHg (pH 7.4) to 80 mmHg (pH 7.15) at a constant PO_2 (200 mmHg), as has been previously reported for the intact *in vitro* CB preparation[27].

REVIEWERS' COMMENTS:

Reviewer #1 (Remarks to the Author):

Thank you for your responses to the critique.
The manuscript is improved and the treatment groups and the rationale for the groups are more clearly delineated.

I have no additional concerns other to say if the carotid bodies are a discrete structure, then the carotid bodies from several adult animals can be pooled to obtain sufficient amount of protein to perform western blots.

Reviewer #2 (Remarks to the Author):

The revised Ms is suitable for publication. Authors answer most of the question raised by this Reviewer

Reviewer #3 (Remarks to the Author):

The manuscript was enormously improved in terms of clarity and rationale.
The authors have answered to almost all the questions raised by this reviewer. Nevertheless, I have to state that although I agree that the amount of carotid bodies needed to measure ATP is high, I do not agree in full with the sentence: afferent discharge from the carotid body is the most sensitive assay for exocytotic release of ATP. There are multiple neurotransmitters being release from the carotid body contributing to set the carotid sinus nerve afferent discharge and therefore differences in the balance of these neurotransmitters may contribute to different afferent responses.

Also, I was unable to find the supplementary data file and this file is key to evaluate some of the requested changes.

Rebuttal to Reviewer comments

REVIEWERS' COMMENTS:

Reviewer #1 (Remarks to the Author):

Thank you for your responses to the critique.
The manuscript is improved and the treatment groups and the rationale for the groups are more clearly delineated.

I have no additional concerns other to say if the carotid bodies are a discrete structure, then the carotid bodies from several adult animals can be pooled to obtain sufficient amount of protein to perform western blots.

Reviewer #2 (Remarks to the Author):

The revised Ms is suitable for publication. Authors answer most of the question raised by this Reviewer

Reviewer #3 (Remarks to the Author):

The manuscript was enormously improved in terms of clarity and rationale.
The authors have answered to almost all the questions raised by this reviewer.
Nevertheless, I have to state that although I agree that the amount of carotid bodies needed to measure ATP is high, I do not agree in full with the sentence: afferent discharge from the carotid body is the most sensitive assay for exocytotic release of ATP. There are multiple neurotransmitters being release from the carotid body contributing to set the carotid sinus nerve afferent discharge and therefore differences in the balance of these neurotransmitters may contribute to different afferent responses.

Also, I was unable to find the supplementary data file and this file is key to evaluate some of the requested changes.

Rebuttal to Reviewer 3

1. *I have to state that although I agree that the amount of carotid bodies needed to measure ATP is high, I do not agree in full with the sentence: afferent discharge from the carotid body is the most sensitive assay for exocytotic release of ATP. There are multiple neurotransmitters being release from the carotid body contributing to set the carotid sinus nerve afferent discharge and therefore differences in the balance of these neurotransmitters may contribute to different afferent responses.*

We have revised the mauscript to address this poir. See page 16, paragraph 2, line 255 to page 18 first line. This now reads:

Thus, LKB1 may maintain in an expression-dependent manner the capacity for ATP synthesis within most cells, including therein carotid body type I cells where ATP deficiency might ultimately impact on the capacity for uptake of ATP by synaptic vesicles and/or exocytotic release of ATP. This view gains indirect support from our finding that *Lkb1* deletion, but not hypomorphic expression of LKB1, showed signs of reducing basal afferent discharge during normoxia, while peak afferent discharge was mildly attenuated by hypomorphic expression of LKB1 (~50%) and virtually abolished by *Lkb1* deletion. Further indirect support is provided by a previous study on rat carotid bodies, which showed that exocytotic release of adenosine represents the principal transmitter contributing to carotid body afferent discharge during mild hypoxia, while ATP acts as the principal transmitter driving afferent discharge during severe hypoxia (i.e., peak discharge frequency) [44].
